# Efficient PET Glycolysis with Suppressed Diethylene Glycol Formation and Beneficial Residue Effects Using an Organic Phosphonate Catalyst

**DOI:** 10.3390/molecules31122160

**Published:** 2026-06-19

**Authors:** Xin-Yu Hao, Xing Cao, Yan-Peng Ni

**Affiliations:** Institute of Functional Textiles and Advanced Materials, College of Textiles & Clothing, Qingdao Key Laboratory of Flame-Retardant Textile Materials, Shandong Key Laboratory of Polymeric Materials Recycling and Upcycling, National Engineering Research Center for Advanced Fire-Safety Materials D & A (Shandong), Qingdao 266071, China

**Keywords:** glycolysis, side reaction suppression, recycled PET, chemical recycling, diethylene glycol

## Abstract

Glycolysis of poly(ethylene terephthalate) (PET) offers a promising route for chemical recycling, yet conventional homogeneous catalysts often suffer from low selectivity, severe side reactions (especially diethylene glycol, DEG formation), and detrimental metal residues that compromise the quality of recycled products. To address these challenges, we herein develop dipotassium phenylphosphonate (PPOA-K) as an efficient homogeneous catalyst for PET glycolysis. Under optimized conditions (1 wt% catalyst, 197 °C, EG/PET mass ratio 3:1, 90 min, atmospheric pressure), PPOA-K achieves 100% PET depolymerization and a high BHET yield of 86.0%, and the reaction follows apparent first-order kinetics with an activation energy of 70.3 kJ·mol^−1^. Beyond its high catalytic activity, PPOA-K effectively suppresses the acid-catalyzed etherification of ethylene glycol to DEG, a common side reaction that reduces monomer purity and degrades recycled polyester properties. Remarkably, the trace amount of PPOA-K remaining in the recovered BHET (17.3 ppm) is not detrimental; instead, it continues to inhibit DEG formation during repolymerization and acts as a thermal stabilizer, improving the melting point and thermal stability of recycled PET. The advantages of PPOA-K are further demonstrated in a partial (in situ) glycolysis–repolymerization process, where it reduces the DEG content in the final rPET to 1.78% (vs. 2.25% for conventional Zn(OAc)_2_), yielding rPET with a higher melting point, higher crystallinity, and better color. This work demonstrates that dipotassium phenylphosphonate uniquely combines high catalytic activity, side reaction suppression, and beneficial residue effects, offering a new catalyst design strategy for high-quality PET recycling.

## 1. Introduction

Poly(ethylene terephthalate) (PET), owing to its excellent chemical stability, mechanical properties, and processability, has become one of the most widely used polymer materials for beverage bottles, textile fibers, and packaging films, with an annual global production exceeding 80 million tons [1,2]. However, the majority of post-consumer PET waste is either landfilled or incinerated, causing severe environmental pollution and resource waste. The growing awareness of plastic pollution and the depletion of fossil resources have driven the urgent need for efficient and sustainable PET recycling technologies [2,3]. At present, PET recycling methods are broadly divided into mechanical and chemical recycling [1,4,5,6]. Mechanical recycling, which involves melting and reprocessing of PET waste, is simple and cost-effective. However, it often leads to chain scission, the accumulation of impurities, and the deterioration of mechanical properties, thus limiting the application of the recycled product to low-value grades [7,8,9,10]. In contrast, chemical recycling enables PET to be depolymerized into monomers or oligomers, which can then be purified and repolymerized to produce high-quality recycled PET, enabling a closed-loop circular economy [11,12,13,14].

Among various chemical recycling routes, glycolysis with ethylene glycol (EG) is considered one of the most promising technologies for industrial application because of its relatively mild reaction conditions, the low volatility of the depolymerizing agent, and the fact that the resulting monomer, bis(2-hydroxyethyl) terephthalate BHET, can be directly repolymerized in one step, thereby shortening the overall recycling process [15,16]. Since the glycolysis of PET proceeds extremely slowly in the absence of a catalyst, the catalyst plays a decisive role in reducing the reaction barrier, enhancing depolymerization efficiency, and improving the BHET yield [16,17,18]. Existing catalytic systems for PET glycolysis are broadly classified as homogeneous and heterogeneous catalysts [19,20]. Heterogeneous catalysts can be recovered by filtration, but their catalytic efficiency is often limited by mass transfer and the poor accessibility of active sites. Moreover, relatively high catalyst loadings are typically required to achieve satisfactory depolymerization [21,22]. In contrast, homogeneous catalysts can be uniformly dispersed in the reaction mixture and typically achieve high catalytic efficiency even at low loadings, making them a research focus [19,23,24]. However, homogeneous catalysts also face challenges arising from difficult separation, which leads to catalyst residue and may in turn promote side reactions, thereby affecting the quality of recycled polyester.

Conventional homogeneous catalysts, especially Lewis acid metal salts such as Zn(OAc)_2_, Mn(OAc)_2_ and titanates, have several inherent drawbacks. These metal ions possess high Lewis acidity, which enables them to activate both the ester bonds of PET (promoting depolymerization) and the hydroxyl groups of EG, thereby lowering the energy barriers for various side reactions, such as the intermolecular etherification of EG to diethylene glycol (DEG) [21,22,25], as well as possible oxidative degradation and thermally induced discoloration. DEG formation is a typical acid-catalyzed nucleophilic substitution reaction that becomes significant at elevated temperatures. It consumes EG, reduces BHET yield and purity, complicates EG recovery, and—when incorporated into the polyester backbone during repolymerization—disrupts chain regularity, lowering the melting point, crystallinity, and thermal stability of recycled PET [26,27]. Moreover, it is difficult to completely remove residual metal ions from BHET and they may continue to catalyze side reactions during the subsequent polycondensation stage, leading to yellowing and deteriorated mechanical properties of the resulting rPET. Various methods, such as using ion exchange resins, are typically used to remove residual metal ions, but their actual removal efficiency is unsatisfactory. For example, when Zn(OAc)_2_ is used as the catalyst, the resulting rPET often exhibits pronounced yellow discoloration. Therefore, the evaluation of catalytic systems for PET glycolysis must consider not only the depolymerization rate and BHET yield but also their ability to suppress a broad range of side reactions (including DEG formation and discoloration) and to avoid the detrimental effects of catalyst residue on the quality of recycled PET [28,29,30]. This need becomes even more critical in partial glycolysis or in situ depolymerization–repolymerization processes, where EG is used in a limited amount and there is no separate EG recovery step. Under such conditions, any DEG formed remains entirely in the system and becomes directly incorporated into the rPET backbone.

Notably, organic phosphonic acids and their salts are commonly used as stabilizers in polyester synthesis and exhibit good compatibility with polyester systems. Therefore, even if trace amounts of an organic phosphonate remain in the regenerated monomer, they are unlikely to adversely affect the preparation of recycled polyester. Instead, they may even contribute to improved properties [31,32]. In addition, in the polyester industry, sodium or potassium carboxylates (e.g., potassium acetate) are frequently used as etherification inhibitors, exploiting the weak basicity of the carboxylate anion to suppress the intermolecular etherification of EG. Inspired by this, we envisioned that an organic phosphonate salt with similarly weak basicity could also inhibit etherification while simultaneously catalyzing PET depolymerization, achieving a “two-in-one” function.

**Figure 1:**
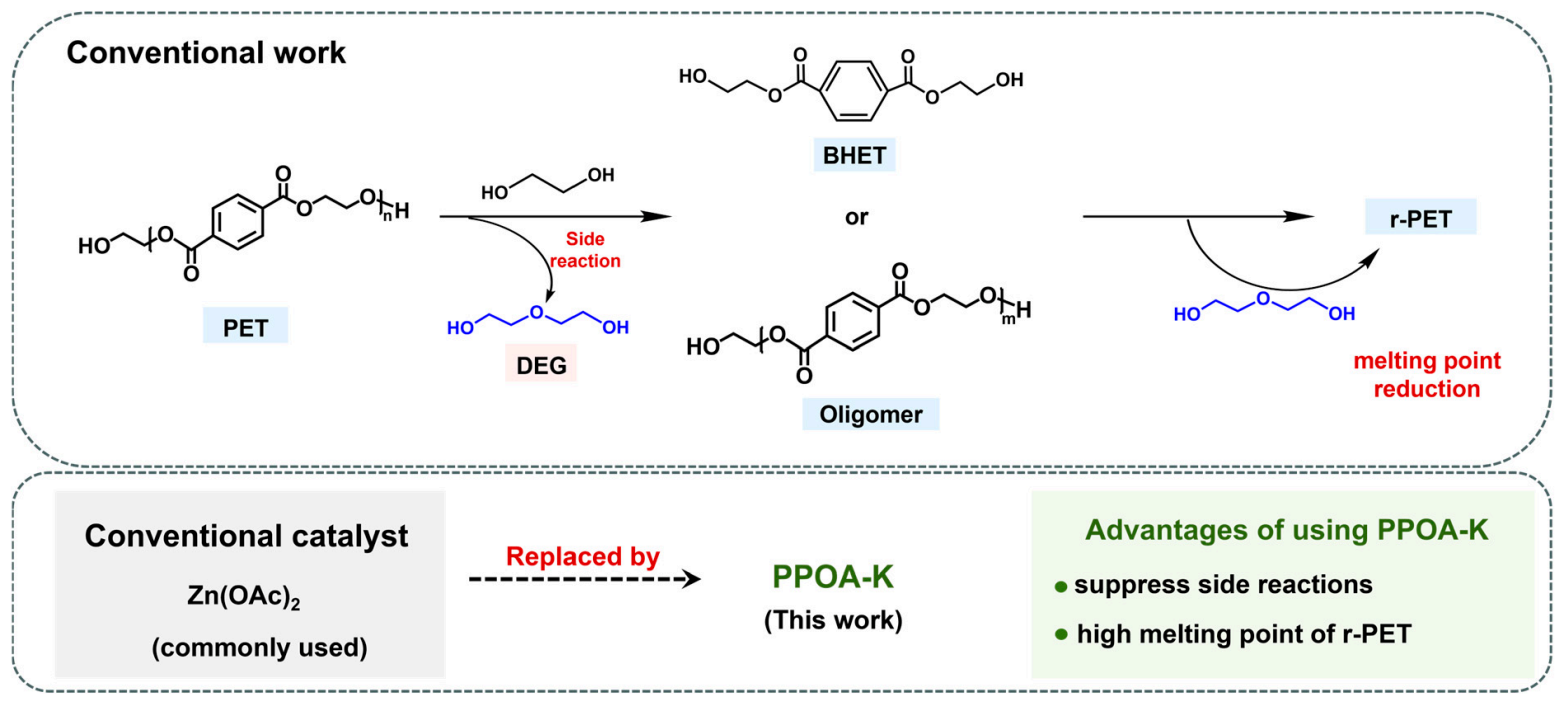
Schematic comparison of Zn(OAc)_2_ and PPOA-K-catalyzed PET glycolysis for r-PET preparation.

Based on the above concept, as shown in Figure 1, we developed dipotassium phenylphosphinate, named PPOA-K, as an organic phosphonate catalyst. PPOA-K is a homogeneous catalyst that dissociates into K^+^ and the phenylphosphonate dianion (C_6_H_5_PO_3_^2−^) in EG. The K^+^ cation acts as a mild Lewis acid to activate the PET carbonyl group, while the anion activates EG via hydrogen bonding. Meanwhile, the weak basicity of the phosphinate anion creates a microenvironment unfavorable for the acid-catalyzed etherification of EG, thus suppressing DEG formation without compromising transesterification efficiency. Using PPOA-K as the catalyst, we systematically optimized the reaction conditions for the complete glycolysis of PET, achieving excellent depolymerization efficiency and BHET yield, and performed kinetic and mechanistic studies. The influence of the recovered monomer on the quality of recycled polyester was evaluated through thermal properties, crystallinity, and colorimetry. Furthermore, PPOA-K was applied to a partial (in situ) glycolysis–repolymerization process, where it significantly suppressed DEG formation, and the resulting rPET exhibited superior thermal properties and color compared to that obtained with the conventional Zn(OAc)_2_ catalyst. This work demonstrates that dipotassium phenylphosphonate uniquely combines high catalytic activity, effective side reaction suppression, and beneficial residue effects, offering a new catalyst design strategy for the efficient depolymerization of PET and the production of high-quality recycled polyester.

## 2. Experimental Section

### 2.1. Materials

Fiber-grade PET chips were obtained from Hengli Chemical Fiber (Suzhou, China). Ethylene glycol (EG, 99.8%), phenylphosphonic acid (PPOA, 99.8%), anhydrous potassium carbonate (99.8%), and ethanol (99.8%) were purchased from Sinopharm Chemical Reagent Co., Ltd. (Shanghai, China).

### 2.2. Preparation of the Potassium Phenylphosphinate PPOA-K

**Scheme 1:**
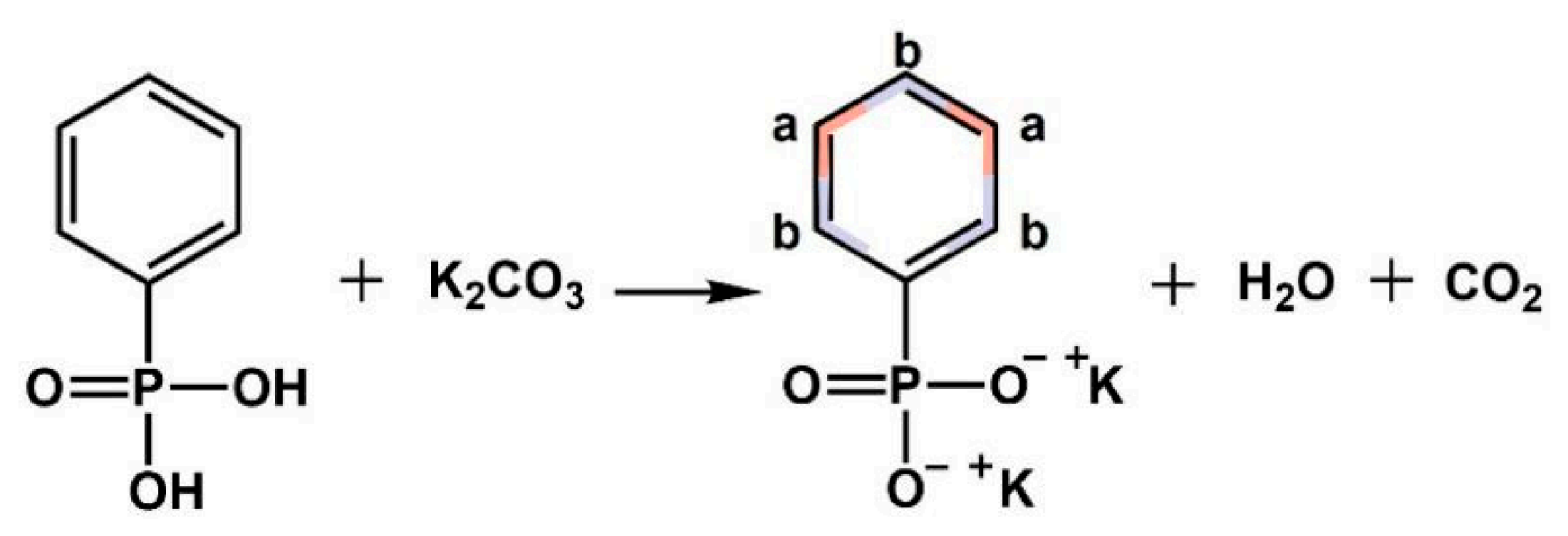
The synthesis of PPOA-K.

A solution of phenylphosphonic acid (31.62 g, 0.20 mol) in ethanol (100 mL) was placed in a three-necked flask. The mixture was heated to 70 °C, and then anhydrous potassium carbonate (33.33 g, 0.201 mol) was added. The reaction was maintained at 70 °C for 30 min. After completion, the mixture was cooled, resulting in the formation of a substantial amount of white precipitate. The reaction mixture was then filtered, and the solid obtained was collected, dried, and stored to afford potassium phenylphosphinate (PPOA-K) in 78% yield (Scheme 1).

### 2.3. Complete Glycolysis of PET

Complete glycolysis of PET was carried out using ethylene glycol (EG). Typically, 5.0 g of PET was added into a reactor containing a predetermined amount of EG. The reactor, equipped with a condenser and a thermometer, was continuously purged with N_2_ to prevent oxidative degradation. After the reactor was immersed in an oil bath and reached the preset temperature, a specified amount of PPOA-K catalyst was added (this moment marked the start of depolymerization). After the designated reaction period, the mixture was hot-filtered to remove any potentially unreacted PET. The filtrate was then diluted with deionized water, heated to 70 °C to dissolve BHET, and hot-filtered again. The final filtrate was kept at 4 °C for 24 h to crystallize BHET. The precipitated crystals were collected and dried as regenerated BHET (rBHET, Solid B).

To ensure uniformity of the feedstock, the PET pellets were ground and sieved, and the 40–60 mesh fraction was collected as PET powder for subsequent glycolysis experiments. The PET depolymerization rate was calculated using Equation (1).

(1) $$\mathrm{PET}\;\mathrm{depolymerization}\;\mathrm{rate}\;(\%)=\frac{m_{0}{-m}_{1}}{m_{0}}\times 100\%$$

where m_0_ is the initial PET mass, and m_1_ denotes the mass of unreacted residue (Solid A).

BHET conversion was determined by high-performance liquid chromatography (HPLC). The cooled reaction liquid was dissolved in 200 mL of methanol/dichloromethane (50:50, *v*/*v*). Using BHET standards as external standards, a calibration curve was constructed to determine the BHET concentration. The BHET conversion (BHET_Y_) is calculated via Equation (2):

(2) $${\mathrm{BHET}}_{Y}=\frac{N_{BHET}}{N_{0}}\times 100\%$$

where *N*_BHET_ is the molar concentration of BHET and *N*_0_ is the molar amount of PET repeating units.

### 2.4. Polymerization of Recycled rBHET

A mixture of virgin BHET or recycled rBHET (50.8 g, 0.20 mol) and antimony trioxide (Sb_2_O_3_, 400 ppm relative to BHET) was charged into a 250 mL custom-built reactor equipped with a heating unit, a water separator, mechanical stirring, and a vacuum system. The reactor was slowly purged with nitrogen. The mixture was stirred at approximately 200 °C for 10 min to ensure complete melting of rBHET. After the reaction mixture became homogeneous, the nitrogen purge was stopped and the vacuum system was turned on. During evacuation, the temperature was gradually raised to 250 °C, and prepolymerization was carried out for 0.5 h under low vacuum. The pressure was then further reduced to a high-vacuum regime (P < 60 Pa), while the temperature was increased to 280 °C, and polycondensation was continued for 2.5 h. Once the melt reached the target viscosity, the reaction was terminated and allowed to cool to room temperature. Following this procedure, virgin PET and recycled rPET_1_ were obtained.

### 2.5. Partial Glycolysis–Repolymerization Route for PET Recycling

The partial glycolysis of PET was performed using a small amount of EG, the dosage of which was far below the EG content inherently present in the PET polymer chain. PET and EG were charged into a reactor, followed by the addition of 0.2 wt% catalyst (relative to PET). The reaction was carried out at 240 °C for 1 h, yielding a partially depolymerized product, consisting mainly of oligomers.

The resulting product was directly transferred to repolymerize without any separation or purification. Prepolymerization was conducted under low vacuum for 0.5 h. The temperature was then raised to 280 °C, and the pressure was further reduced to high vacuum (P < 60 Pa). Polycondensation was continued for 2.0 h. Once the melt reached the target viscosity, the reaction was terminated, and the product was cooled to room temperature. This procedure afforded recycled PET (rPET_2_).

### 2.6. Characterization

Depolymerization products at different reaction stages were quantitatively analyzed using an Agilent 1000-series HPLC, from which BHET conversion was calculated. Upon completion of the depolymerization reaction, the reactor was immediately immersed in an excess of cold water to rapidly quench the reaction. An appropriate aliquot of the reaction mixture was then withdrawn using a micropipette, transferred to a 25 mL volumetric flask, and diluted to volume with a methanol/dichloromethane mixture (50/50, *v*/*v*). The resulting solution was subjected to HPLC analysis.

The HPLC conditions were as follows: a C18 reversed-phase chromatographic column was used; UV detection was performed at 254 nm; the mobile phase was composed of acetonitrile and water; and gradient elution was programmed as follows: 0–10 min, acetonitrile increased from 20% to 80%; 11–12 min, acetonitrile increased from 80% to 95%; and 13–15 min, acetonitrile returned from 95% to 20%. Samples were injected using an autosampler with an injection volume of 3 μL. The column temperature was maintained at 40 °C, and the flow rate was 1.0 mL min^−1^.

The elemental composition and chemical states of PPOA-K were characterized by X-ray photoelectron spectroscopy (XPS) using an ESCALAB Xi+ X-ray photoelectron spectrometer (Thermo Fisher Scientific, Waltham, MA, USA).

^1^H, ^13^C, and ^31^P NMR spectra of the relevant compounds were acquired on a JNM-ECZ600R/S1 spectrometer (JEOL, Tokyo, Japan). Deuterium oxide (D_2_O) was used as the solvent for PPOA−K, deuterated dimethyl sulfoxide (DMSO-*d*_6_) for other small molecules (e.g., BHET), and deuterated trifluoroacetic acid (TFA-d) for PET samples.

FTIR spectra were collected on a NicoletiS50 spectrometer (Thermo Fisher Scientific, Waltham, MA, USA) using the KBr pellet method.

The thermal stability of regenerated PET was evaluated using a TGA5500 thermogravimetric analyzer (TA Instruments, New Castle, DE, USA) under nitrogen and air atmospheres. Measurements were conducted from 40 to 750 °C at a heating rate of 10 °C min^−1^.

The melting and crystallization behavior of regenerated PET was characterized by differential scanning calorimetry (DSC) using a DSC2500 calorimeter (TA Instruments). Approximately 5 mg of sample was first heated rapidly to 280 °C and held for 3 min, then cooled to 0 °C at 10 °C min^−1^ and held for 3 min, and finally reheated to 280 °C at 10 °C min^−1^.

## 3. Results and Discussion

### 3.1. Characterization of the Phenylphosphinate–Salt Catalyst PPOA−K

**Figure 2:**
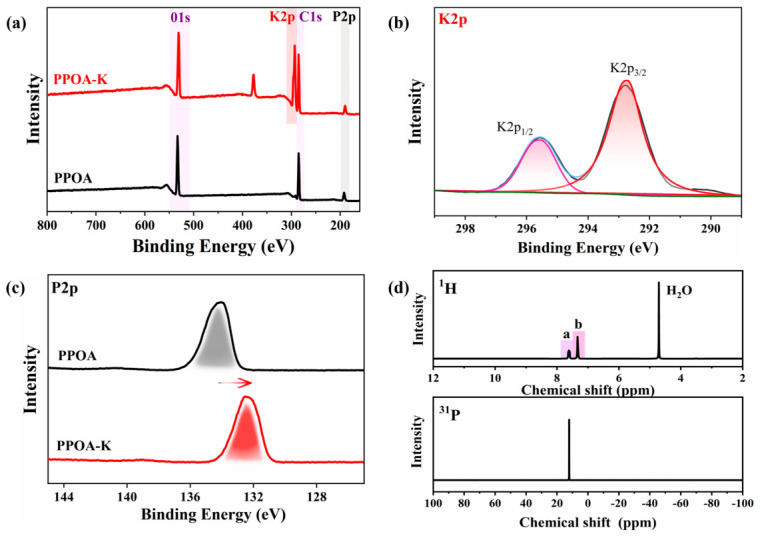
(**a**) XPS survey spectra of PPOA and PPOA−K; (**b**) high-resolution K2p XPS spectra of PPOA−K; (**c**) high−resolution P2p XPS spectra of PPOA and PPOA−K; (**d**) ^1^H NMR and ^31^P NMR spectra of PPOA−K.

The chemical structure of PPOA−K, a phenylphosphinate–salt catalyst, was characterized by XPS and NMR, and the results are presented in Figure 2. The XPS survey spectrum of PPOA−K (Figure 2a) shows signals for C, O, and P, along with a distinct K2p signal. In the high-resolution K2p spectrum (Figure 2b), two well-resolved spin–orbit components assigned to K2p_1/2_ and K2p_3/2_ appear at binding energies of 295.6 and 292.7 eV, respectively, confirming the presence of potassium [33]. Figure 2c compares the P_2_p spectra of PPOA and PPOA−K. The P_2_p binding energy shifts from 134.2 eV for PPOA to 132.4 eV for PPOA−K, indicating conversion of the phosphonic acid moiety to the corresponding potassium phenylphosphinate [33,34]. The ^1^H and ^31^P NMR spectra of PPOA−K are shown in Figure 2d. In the ^1^H NMR spectrum, resonances at 7.61 and 7.34 ppm are assigned to aromatic protons on the phenyl ring, with an integral ratio of 2:3. The ^31^P NMR spectrum exhibits a single resonance at 12.17 ppm [34,35]. Collectively, these results confirm the successful preparation of the ionized phosphate–salt catalyst PPOA−K.

### 3.2. Catalytic Performance of PPOA−K in PET Glycolysis

**Figure 3:**
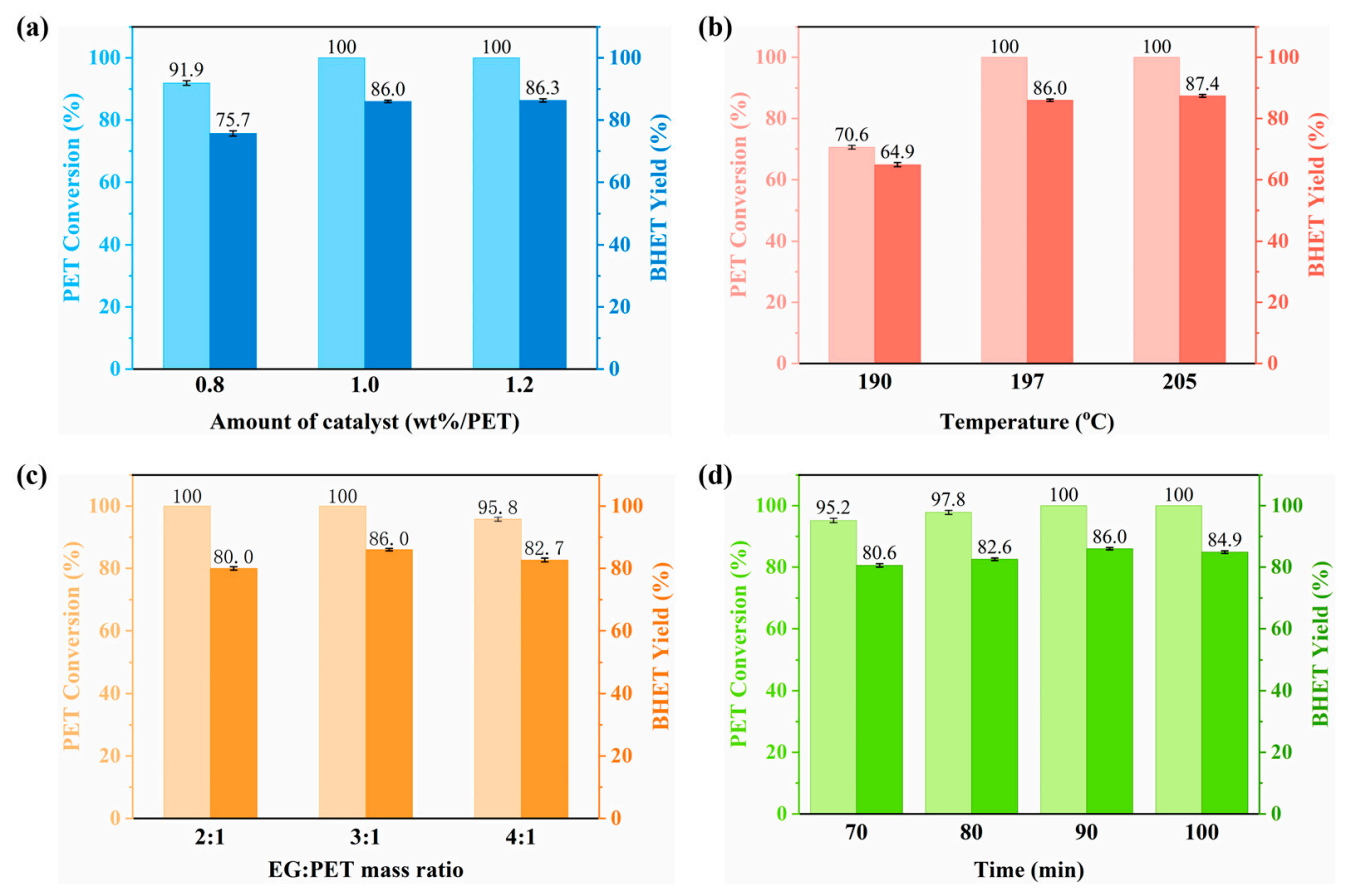
The effects of different reaction conditions on the PET depolymerization rate and BHET conversion catalyzed by PPOA−K: (**a**) catalyst loading, (**b**) reaction temperature, (**c**) EG dosage, and (**d**) reaction time.

The catalytic performance of PPOA−K in the glycolysis of PET was systematically evaluated by examining the effects of catalyst dosage, reaction temperature, EG/PET mass ratio, and reaction time on PET depolymerization efficiency and BHET yield. The results are presented in Figure 3.

The influence of catalyst loading was first investigated at an EG/PET mass ratio of 3:1, a reaction temperature of 197 °C, and a reaction time of 90 min. As shown in Figure 3a, the PET depolymerization rate and BHET yield increased sharply when the catalyst loading was raised from 0.8 wt% to 1.0 wt%. Further increasing the loading to 1.2 wt% gave only a marginal improvement. Thus, the optimal catalyst loading was determined to be 1.0 wt%.

Next, the effect of reaction temperature was examined under the same conditions (1.0 wt% PPOA−K, EG/PET = 3:1, 90 min). Raising the temperature from 190 °C to 197 °C increased the PET depolymerization rate from 70.6% to 100%, with a BHET yield of 86.0% at 197 °C. Elevated temperatures provide additional energy to overcome the activation barrier and enhance effective collisions. Since 197 °C is close to the boiling point of EG and allows for atmospheric pressure operation, it was chosen as the optimal temperature. The EG/PET mass ratio also significantly affected the depolymerization performance. Under the same conditions (1.0 wt% PPOA−K, 197 °C, 90 min), a ratio of 3:1 gave the best depolymerization rate and BHET yield.

Finally, the influence of reaction time was investigated after determining the optimal catalyst loading, temperature, and EG/PET ratio. As shown in Figure 3d, complete depolymerization (100%) was achieved at 90 min. Prolonging the reaction time beyond 90 min, however, led to a decrease in BHET conversion. This is likely due to the dynamic equilibrium between BHET and oligomers. After PET is fully depolymerized, the continuous presence of the catalyst may promote the conversion of BHET into dimers, thereby reducing the BHET yield. Therefore, appropriate control of the reaction time not only avoids energy waste but also maximizes BHET conversion.

Overall, PPOA−K enables efficient PET glycolysis under relatively mild atmospheric pressure conditions within a relatively short reaction time, affording a high BHET yield.

### 3.3. Kinetic Study and Mechanism of PET Glycolysis Catalyzed by PPOA−K

**Figure 4:**
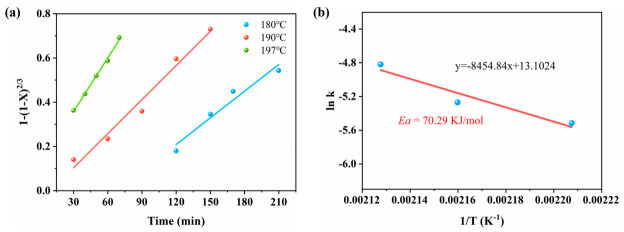
(**a**) The effect of temperature on the glycolysis rate of PET. Reaction conditions: PET (5.0 g), EG (15.0 g), 1 wt% PPOA−K catalyst, under atmospheric pressure; (**b**) an Arrhenius plot of the rate constants for PET glycolysis.

To clarify the mechanism of PET depolymerization catalyzed by the PPOA−K, the kinetics of PET glycolysis were investigated. PET glycolysis in ethylene glycol is governed by multiple factors, including the EG−to−PET mass ratio, catalyst loading, the morphology and particle size of the PET feedstock, and the stirring rate. In the present system, PPOA−K is soluble in EG, whereas PET is insoluble. Therefore, PET glycolysis can be regarded as a solid–liquid two−phase reaction occurring at the PET surface. Because small PET particles can be reasonably approximated as spheres, experimental data obtained using 40–60 mesh PET powder were employed to simplify the kinetic analysis. Given the complexity of this heterogeneous system, several kinetic models have been proposed, such as the shrinking core model, the random scission model, and other mathematical formulations [36]. Based on these assumptions, the kinetic model was preliminarily assumed to follow the shrinking core model, with the following governing equation:

(3) $$k^{\prime }t\;=\;1\;-\;{(1\;-\;x)}^{2/3}$$

where $k^{\prime }$ is the apparent rate constant and x is the BHET conversion. BHET conversion over PPOA−K was evaluated at different temperatures and reaction times. Linear regression of the experimental data (Figure 4a) provided the apparent rate constant at each temperature (Table 1).

Furthermore, the activation energy of depolymerization (*E*a) was determined using the Arrhenius equation:

(4) $$\mathrm{Lnk}\;=\;\mathrm{lnA}\;-\;\frac{\mathrm{Ea}}{\mathrm{RT}}$$

where A is the pre−exponential factor,  R  is the gas constant (8.314 J·K^−1^·mol^−1^), and T is the absolute temperature. As shown in Figure 4b, the Arrhenius plot gave a linear fit, yielding an apparent activation energy of *E*_a_ = 70.29 kJ·mol^−1^. This result indicates that the PPOA−K−catalyzed glycolysis of PET follows an apparent first−order kinetic model. This kinetic evaluation provides a theoretical basis for elucidating the intrinsic mechanism of the reaction and offers guidance for optimizing subsequent reaction conditions.

**Figure 5:**
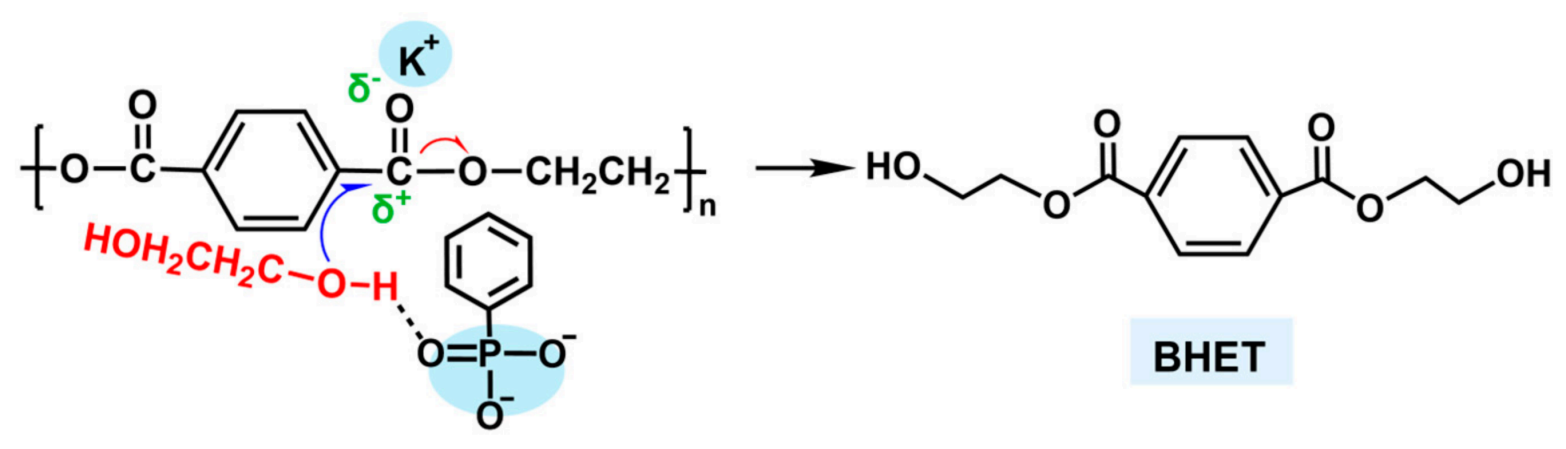
Proposed mechanism for PPOA-K−catalyzed depolymerization of PET.

The glycolytic depolymerization of PET is essentially a transesterification process [37]. Based on the kinetic analysis and the structural characteristics of PPOA−K, a synergistic mechanism for its catalysis of PET glycolysis is proposed, as illustrated in Figure 5.

PPOA−K dissociates into K^+^ and the phenylphosphinate anion (PhP(O)(O^−^)H) in EG. In the catalytic cycle, K^+^ acts as a Lewis acid by coordinating to the carbonyl oxygen of the PET ester bond, polarizing the C=O bond and making the carbonyl carbon more electrophilic, thereby lowering the energy barrier for nucleophilic attack [38,39,40]. Meanwhile, the phosphinate anion forms strong hydrogen bonds with the hydroxyl proton of EG through its P=O or P−O^−^ groups, polarizing the O−H bond and enhancing the nucleophilicity of the EG oxygen.

Subsequently, the activated EG attacks the K^+^-activated carbonyl carbon, forming a transition state (likely a four- or six-membered ring) involving PET, EG, and the catalyst [39]. This transition state facilitates the cleavage of the ester bond, generating BHET and oligomers while regenerating the catalyst.

### 3.4. Suppression of Diethylene Glycol (DEG) Side Reaction

During PET glycolysis, EG can undergo an undesirable intermolecular etherification side reaction to form diethylene glycol (DEG) [41]. This reaction is favored under elevated temperatures, prolonged reaction times, and in the presence of Brønsted or Lewis acid catalysts. The reaction typically proceeds via a nucleophilic substitution mechanism: one EG molecule is activated by protonation (or coordination to a Lewis acid), and then its hydroxyl group is attacked by the hydroxyl group of a second EG molecule, resulting in the formation of a C-O-C ether bond and the elimination of water.

In PET glycolysis recycling, DEG formation has several adverse effects. The formation of DEG consumes EG, which reduces the recovery yield of EG. Furthermore, because DEG and EG have close boiling points (DEG 245 °C, EG 197 °C), they are difficult to separate completely during EG distillation, leading to residual DEG in the recovered EG. When such DEG-contaminated EG is reused in subsequent polymerization, for example in the polycondensation with BHET to produce recycled PET, the DEG units become incorporated into the polymer backbone. This disrupts chain regularity and consequently lowers the melting point, crystallinity, and thermal stability of rPET.

It should be noted that DEG content is itself a key quality control parameter in conventional PET product specifications, whether for virgin or recycled PET, and must be strictly limited. This is especially critical in partial glycolysis or in situ depolymerization–repolymerization processes, where the amount of EG used is not small, there is no separate EG recovery step and the reaction product is directly subjected to polycondensation. Under such conditions, any DEG generated remains entirely in the system and is incorporated into the polymer backbone, having a much more direct and severe impact on the properties of rPET. Therefore, suppressing the DEG side reaction during the recycling process is essential for producing high-quality recycled PET that meets industry standards and serves as an important criterion for evaluating the overall performance of catalysts.

**Figure 6:**
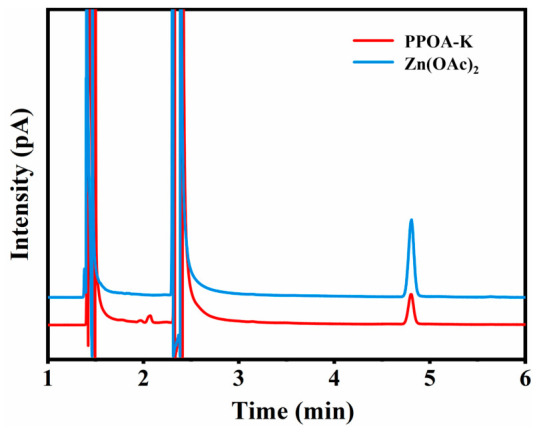
Gas chromatograms of DEG in different PET glycolysis systems.

The DEG content in the glycolysis products from different catalytic systems was measured by gas chromatography (GC), as shown in Figure 6 and Table 2. Because the PET feedstock itself contains a small amount of DEG comonomer units, the background DEG concentration in the test solution was first determined to be 129.1 mg/L. After high-temperature glycolysis, the DEG concentration in the reaction mixture using the Zn(OAc)_2_ catalyst increased sharply to 286.3 mg/L, approximately 2.2 times the background level. In contrast, the DEG concentration in the system using the PPOA−K catalyst was only 142.1 mg/L, showing only a slight increase from the background.

The suppression of DEG formation by PPOA−K is attributed to the weakly basic microenvironment generated by its dissociation. The intermolecular etherification of EG to form DEG is an acid-catalyzed reaction, which is effectively inhibited under basic conditions. PPOA−K dissociates into potassium cations and phenylphosphinate anions in EG; the latter provides the weak basicity, analogous to the use of sodium or potassium carboxylates as etherification inhibitors in the polyester industry. Experimental data clearly demonstrate that PPOA−K significantly reduces DEG formation.

### 3.5. Structural Characterization and Quality Assessment of r-BHET

**Figure 7:**
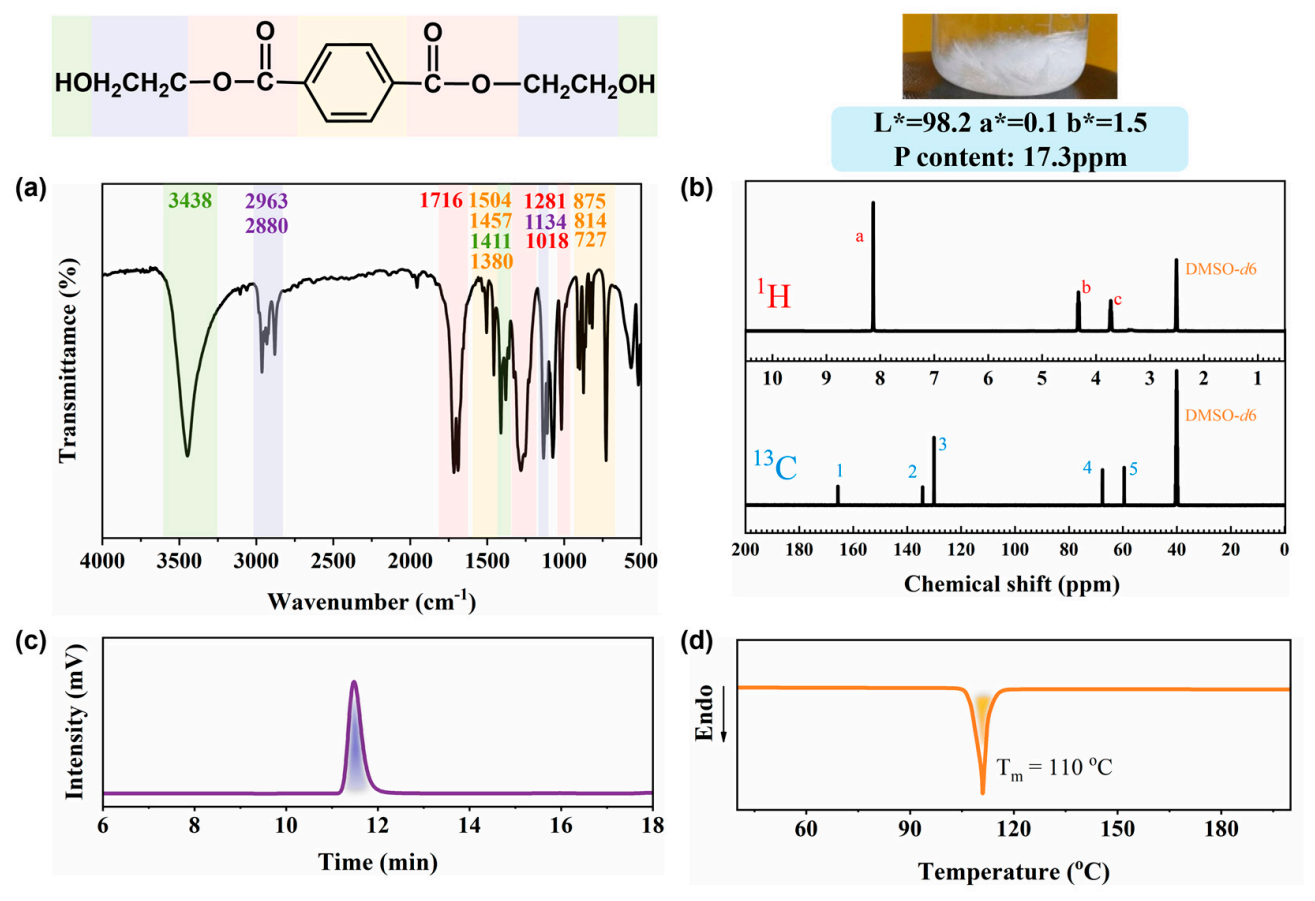
(**a**) FTIR spectrum, (**b**) ^1^H NMR and ^13^C NMR spectra, (**c**) photograph, and (**d**) DSC curve of recovered monomer obtained from PET glycolysis catalyzed by PPOA−K.

To comprehensively evaluate the quality of the r−BHET monomer obtained from PPOA−K−catalyzed glycolysis of PET under the optimized reaction conditions, the product was characterized by FTIR, NMR, DSC, HPLC, ICP-OES and colorimetry. The corresponding results are shown in Figure 7.

As shown in the FTIR spectrum of r-BHET (Figure 7a), the strong absorption band at 3438 cm^−1^ corresponds to the stretching vibration of O-H, while the band at 1411 cm^−1^ is assigned to the in-plane bending vibration of C-O-H, indicating the presence of hydroxyl groups [42]. The CH_2_ asymmetric and symmetric stretching vibrations appear at 2963 and 2880 cm^−1^, respectively, and the CH_2_ rocking vibration at 1134 cm^−1^, confirming the presence of methylene groups. The band at 1716 cm^−1^ is assigned to the stretching vibration of the ester carbonyl C=O bond, while the peaks at 1281 and 1018 cm^−1^ correspond to the C-O stretching vibrations related to the carbonyl group and the alkyl group, respectively, indicating the presence of ester functional groups in BHET. In addition, the absorption peaks at 1504, 1457, and 1380 cm^−1^ are attributed to the skeletal vibrations of the aromatic ring, while the peaks at 875, 814, and 727 cm^−1^ correspond to the out-of-plane C-H bending vibrations of the benzene ring, confirming the presence of the aromatic ring [42,43].

The ^1^H and ^13^C NMR spectra (Figure 7b) further confirm the structure of BHET. In the ^1^H NMR spectrum, the signal at 8.13 ppm is assigned to the four protons on the benzene ring. The peak at 4.99 ppm corresponds to the protons of the hydroxyl groups [42,44,45]. The methylene protons adjacent to the ester groups resonate at 4.33 ppm, and those adjacent to the hydroxyl groups at 3.74 ppm. In the ^13^C NMR spectrum, the peak at 165.71 ppm is assigned to the ester carbonyl carbon attached to the benzene ring. The signal at 134.28 ppm corresponds to the aromatic carbon bonded to the ester group, and the peak at 130.06 ppm to the remaining four aromatic carbons. The methylene carbon adjacent to the ester group appears at 67.57 ppm, and that adjacent to the hydroxyl group at 59.51 ppm. No extraneous signals are observed, indicating high purity of the recovered BHET.

The HPLC chromatogram shown in Figure 7c further confirms the high purity of the recovered BHET, as only a sharp single peak appears at 11.8 min. The DSC curve (Figure 7d) shows a single endothermic peak at 110.6 °C, corresponding to the melting point of BHET. All these characterization results are consistent with literature data for BHET. Thus, the main product of PPOA−K−catalyzed PET glycolysis is indeed BHET, and the catalytic process does not adversely affect the structure of the recovered monomer. The color of the recovered BHET was measured using a colorimeter in the CIE Lab color space. The measured values were L* = 98.2, a* = 0.1, and b* = 1.5. The high L value indicates excellent whiteness, while the near-zero a and low b values suggest negligible red–green and yellow–blue color components.

ICP-OES analysis revealed a residual phosphorus content of 17.3 ppm in the recovered BHET, indicating that only a trace amount of the PPOA−K catalyst remains. Notably, this trace residue is not necessarily detrimental. Since PPOA−K itself is capable of suppressing the EG etherification side reaction, the small amount of catalyst remaining in BHET can continue to inhibit DEG formation during subsequent polycondensation to produce rPET. This helps to lower the DEG content in the recycled polyester, thereby improving the melting point, crystallinity, and thermal stability of rPET. This further highlights a unique advantage of the PPOA−K catalyst.

### 3.6. Influence of Catalyst Residues in rBHET on Performance of Recycled PET

To investigate the effect of this trace residue on the properties of recycled PET, we further carried out a series of performance tests on the rPET obtained by polymerizing this rBHET.

**Figure 8:**
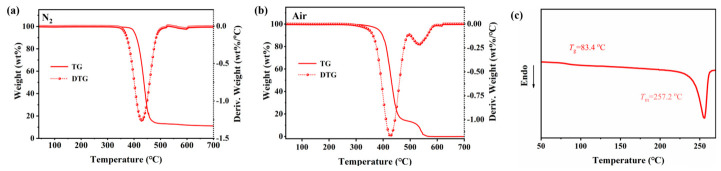
TGA curves (**a**,**b**) and DSC curves (**c**) of rPET_1_.

Figure 8 presents the TGA and DSC curves of rPET and Table 3 summarizes the TGA data. Compared with virgin PET, both the initial decomposition temperature (T_5%_) and the maximum thermal decomposition temperature (T_dmax_) of rPET were improved. Under a nitrogen atmosphere, the T_5%_ and T_dmax_ of PET were 391.6 °C and 429.1 °C, respectively, whereas those of rPET_PPOA−K_ increased to 400.1 °C and 431.7 °C, respectively. Under an air atmosphere, the improvement in thermal stability was more pronounced. Specifically, the T_5%_, T_dmax1_, and T_dmax2_ of PET were 355.7 °C, 423.1 °C, and 517.1 °C, respectively, while those of rPET_PPOA−K_ increased to 383.4 °C, 426.7 °C, and 533.9 °C, respectively.

The ether bond in the diethylene glycol (DEG) segment is thermally unstable and prone to cleavage at high temperatures, leading to degradation of the PET molecular chain. The improved thermal stability of rPET may be attributed to two factors. On the one hand, the residual PPOA−K in rBHET suppresses DEG formation during repolymerization, thereby reducing the number of unstable ether bonds in the polymer backbone. On the other hand, the phosphonate itself can act as a thermal stabilizer, directly enhancing the thermal decomposition temperature of the polyester. The combined effect of these two factors results in higher thermal stability of rPET compared to virgin PET. Moreover, excessive DEG content in PET disrupts chain regularity and weakens intermolecular interactions, resulting in a lower melting temperature. In this study, the melting temperature of rPET increased to 257.2 °C, which is higher than the melting point of typical commercial PET. The DSC results further indicate that rPET contains fewer ether bonds, demonstrating that the residual PPOA−K in rBHET has a positive effect on the repolymerization process for preparing rPET.

### 3.7. Verification in an In Situ Glycolysis–Repolymerization System

In the complete glycolysis process described above, PPOA−K has demonstrated excellent catalytic activity and DEG suppression capability. To further verify the applicability of this catalyst to different recycling processes, we applied it to an in situ glycolysis–repolymerization system (i.e., a partial glycolysis process). In this process, a small amount of ethylene glycol is used for micro-glycolysis of PET, and the reaction product is directly subjected to polycondensation without separation to produce rPET_2_. A zinc acetate (Zn(OAc)_2_) system was used as a reference. The thermal properties of the two systems were compared.

**Figure 9:**
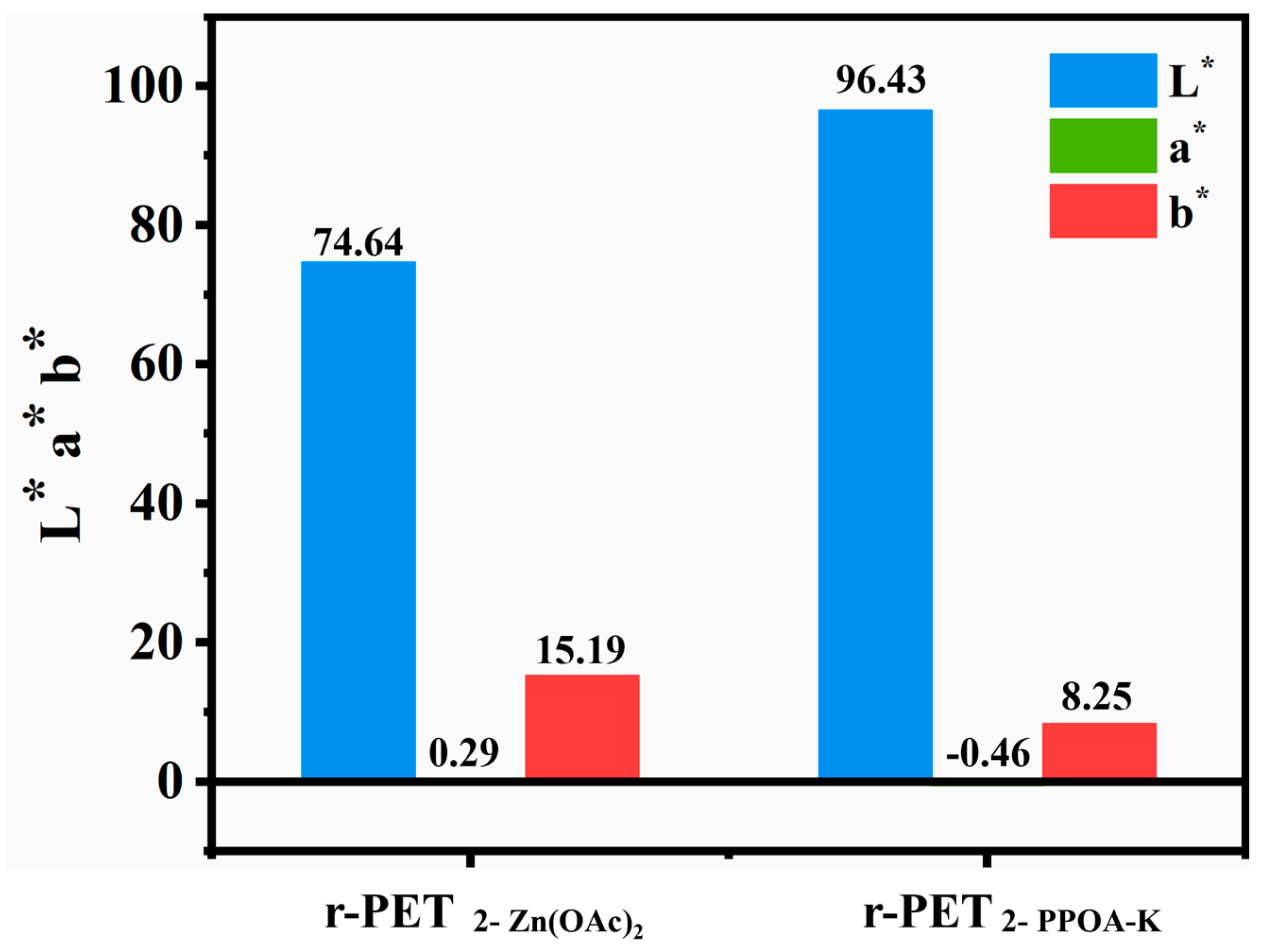
The Lab value of PET partial glycolysis–repolymerization products catalyzed by anhydrous Zn(OAc)_2_ and PPOA−K.

As shown in Figure 9, in both the PPOA−K− and Zn(OAc)_2_−catalyzed in situ systems, PET was partially depolymerized within 1 h at 240 °C and the resulting oligomer mixtures directly afforded rPET_2_ with high intrinsic viscosity. Notably, the polyester regenerated using PPOA−K as the catalyst exhibited superior color appearance, whereas the Zn(OAc)_2_ system displayed slight yellowing. The colorimetric analysis revealed the following values: for the PPOA−K system, L* = 96.43, a* = −0.46, and b* = 8.25; for the Zn(OAc)_2_ system, L* = 74.64, a* = 0.29, and b* = 15.19.

**Figure 10:**
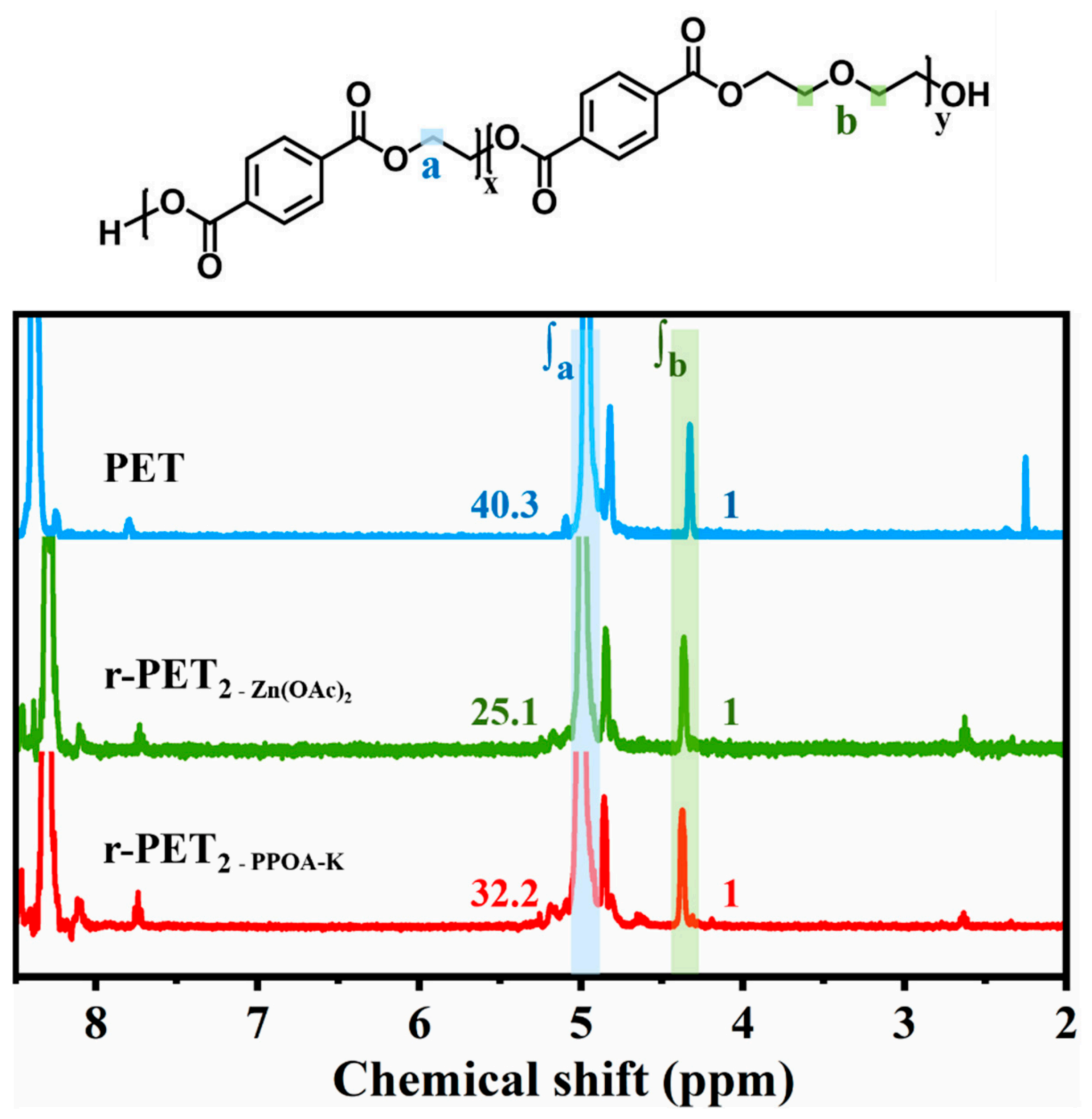
^1^H NMR characterization of PET partial glycolysis–repolymerization products catalyzed by anhydrous Zn(OAc)_2_ and PPOA-K (“∫” indicates the integration of the corresponding peak).

**Figure 11:**
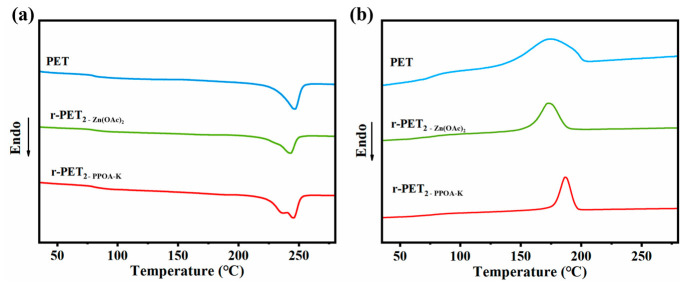
DSC characterization of PET partial glycolysis–repolymerization products catalyzed by anhydrous Zn(OAc)_2_ and PPOA-K: (**a**) heating curves; (**b**) cooling curves.

More importantly, since there is no separate EG recovery step in this process, any by-product DEG remains entirely in the reaction system. As shown in Figure 10, ^1^H NMR analysis indicated that the content of diethylene glycol segments in the recycled rPET was higher than that in virgin PET for both catalysts, confirming that the recycling process inevitably leads to an increase in DEG content. Nevertheless, the DEG content in the PPOA-K system was significantly lower than that in the Zn(OAc)_2_ system. Quantitative calculation showed that, thanks to the efficient suppression of the etherification reaction by PPOA-K, the final rPET contained only 1.78% DEG comonomer units, much lower than the 2.25% obtained with the Zn(OAc)_2_ reference system. Accordingly, as evidenced by the DSC curves presented in Figure 11 and Table 4, the melting point (245.7 °C) and crystallinity (22.8%) of the rPET obtained from the PPOA-K system were significantly higher than those of the reference system (243.2 °C and 15.7%, respectively).

## 4. Conclusions

In this study, we have developed dipotassium phenylphosphonate (PPOA-K) as an efficient organic phosphonate catalyst for the glycolysis of poly(ethylene terephthalate). Under optimized mild atmospheric conditions, PPOA-K achieves complete PET depolymerization and a high BHET yield. Kinetic analysis confirms an apparent first-order kinetic model, and a synergistic mechanism involving K^+^ activation of the carbonyl group and dianion activation of EG via hydrogen bonding is proposed. A key advantage of PPOA-K is its ability to suppress the acid-catalyzed etherification of EG to DEG, a common side reaction that compromises r-EG purity and recycled polyester quality. This suppression arises from the weakly basic microenvironment created by the phenylphosphonate dianion. Notably, the trace residual PPOA-K in the recovered BHET is not detrimental. Instead, it continues to inhibit DEG formation during repolymerization and acts as a thermal stabilizer, thereby improving the thermal properties of recycled PET. The advantages of PPOA-K are further verified in a partial (in situ) glycolysis–repolymerization process. Compared with the conventional Zn(OAc)_2_ catalyst, PPOA-K significantly reduces DEG formation, yielding rPET with higher melting point, higher crystallinity, and better color. Overall, dipotassium phenylphosphonate uniquely combines high catalytic activity, effective side reaction suppression, and beneficial residue effects, offering a new catalyst design strategy for efficient PET depolymerization and high-quality recycled polyester. This work demonstrates the potential applicability of PPOA-K in both complete and partial glycolysis processes, providing a sustainable solution for PET chemical recycling.

## Figures and Tables

**Table 1:** Reaction rate constants (k) at different reaction temperatures.

Temperature (°C)	Reaction Rate Constant (min^−1^)
180	0.00402
190	0.00514
197	0.00807

**Table 2:** DEG content in different PET glycolysis systems.

	Description	Average DEG Concentration (mg/L)
1	Original PET	129.1
2	Zn(OAc)_2_	286.3
3	PPOA−K	142.1

**Table 3:** TG data of PET and rPET_1_.

Samples	N_2_		Air		
	T_5%_ (°C)	T_dmax_ (°C)	T_5%_ (°C)	T_dmax1_ (°C)	T_dmax2_ (°C)
PET	391.6	429.1	355.7	423.1	517.1
rPET_1_	400.1	431.7	383.4	426.7	533.9

**Table 4:** DSC data and DEG content changes of rPET from partial glycolysis–repolymerization.

Sample	T_g_ (°C)	T_m_ (°C)	T_cc_ (°C)	ΔH_m_ (J/g)	ΔH_cc_ (J/g)	X_c_ (%)	DEG Content (%)
PET	81.3	248.6	180.4	34.3	30.4	21.7	1.43
r-PET_2-Zn(OAc)2_	81.7	243.2	172.7	24.4	22.1	15.7	2.25
r-PET_2-PPOA-K_	81.4	245.7	186.7	34.9	31.9	22.8	1.78

## Data Availability

The original contributions presented in this study are included in the article. Further inquiries can be directed to the corresponding authors.
